# The effects of curcumin on hepatic T2*MRI and liver enzymes in patients with β‐thalassemia major: a double‐blind randomized controlled clinical trial

**DOI:** 10.3389/fphar.2023.1284326

**Published:** 2023-12-18

**Authors:** Aziz Eghbali, Shima Nourigheimasi, Ali Ghasemi, Roghayeh Rahimi Afzal, Neda Ashayeri, Aygin Eghbali, Shokoufeh Khanzadeh, Kazem Ghaffari

**Affiliations:** ^1^ Clinical Research Development Center of Aliasghar Hospital, Iran University of Medical Sciences, Tehran, Iran; ^2^ School of Medicine, Amir Kabir Hospital, Arak University of Medical Sciences, Arak, Iran; ^3^ Department of Biochemistry and Hematology, Faculty of Medicine, Semnan University of Medical Sciences, Semnan, Iran; ^4^ Department of Pediatrics, Amir Kabir Hospital, Arak University of Medical Sciences, Arak, Iran; ^5^ School of Medicine, Tabriz University of Medical Sciences, Tabriz, Iran; ^6^ Department of Basic and Laboratory Sciences, Khomein University of Medical Sciences, Khomein, Iran; ^7^ Student Research Committee, Khomein University of Medical Sciences, Khomein, Iran

**Keywords:** curcumin, liver enzymes, β-thalassemia major, T2∗MRI, aspartate transaminase, randomized clinical trial

## Abstract

**Background:** Curcumin present in turmeric has been considered due to its cancer-preventive features, antioxidant and anti-inflammatory properties. This double-blind, randomized, controlled clinical trial with a reasonable sample size and longer intervention period was conducted to investigate how oral curcumin affected cardiac and hepatic T2*MRI and liver enzymes in patients with β‐thalassemia major.

**Method:** This clinical trial study was conducted on 171 patients over 5 years old. The subjects were randomly divided into a curcumin-treatment group and a placebo group to receive either curcumin capsules twice daily or placebo for 6 months. Patients were examined once a month for 6 months to receive capsules and measure the levels of alanine aminotransferase (ALT), aspartate transferase (AST), alkaline phosphatase (ALP), direct and total bilirubin, ferritin and cardiac and hepatic T2*MRI.

**Result:** There was a significant decrease in levels of AST, ALT, ALP, and bilirubin (direct and total) in the curcumin group compared with the placebo group by the end of the study (*p* < 0.05). The levels of serum ferritin remained unchanged in both groups at the end of the follow‐up period (*p* > 0.05). No significant differences were observed between the curcumin and placebo groups at baseline values or at the end of the study of cardiac and hepatic T2*MRI and serum magnesium.

**Conclusion:** Administration of curcumin has some beneficial effects on liver function by reducing liver enzymes in patients with beta-thalassemia major.

## 1 Introduction

Beta thalassemia has autosomal recessive inheritance and is associated with defects in the beta chain of hemoglobin. The prevalence of beta thalassemia is very high in the Middle East and Southeast Asia (Colah et al., 2010). The frequency of beta-thalassemia varies between 4% and 10% in different states of Iran, and about 10% of the population of Iran are carriers of beta-thalassemia (Hashemizadeh and Noori, 2013). An imbalance in the production of globin chains has been observed in homozygous or mixed heterozygous forms, which is ultimately associated with a decrease in normal hemoglobin production, severe chronic hemolytic anemia, and ineffective hematopoiesis (Weatherall, 2001; Gulbis et al., 2010). Due to the complications mentioned above, patients with β‐thalassemia major need regular blood transfusions from early childhood. Long-term blood transfusion causes iron overload in various tissues and, as a result, heart complications, liver disease and endocrine dysfunction (Gulbis et al., 2010). Anemia in patients with beta-thalassemia intermedia is often mild to moderate, but patients, although less than those with β‐thalassemia major, may still need occasional blood transfusions (Weatherall, 2001; Gulbis et al., 2010).

The deposit of excess iron in the liver cells is one of the main problems with thalassemia. Therefore, it is necessary to use iron chelators to prevent excess iron deposition in various tissues of these patients (Gardenghi et al., 2007). The life expectancy of patients has increased since the discovery of iron-chelating drugs. There are currently three chelating drugs: deferoxamine, deferiprone, and deferasirox. Defroxamine has limited oral absorption and must be administered subcutaneously, intravenously, or intramuscularly, but deferiprone and deferasirox are oral iron chelators and are associated with better patient compliance (Wali et al., 2004; Rachmilewitz and Giardina, 2011). However, none of the two mentioned oral chelators are perfect, and the use of a chelator with high effectiveness is felt as an alternative to deferoxamine or for use in combination therapy (Meerpohl et al., 2012; Weeraphan et al., 2013).

Today, curcumin present in turmeric has been considered due to its cancer-preventive features, antioxidant and anti-inflammatory properties (Milani et al., 2017; Nasseri et al., 2018). Several studies have shown that curcumin may form complexes with various metal ions, especially iron, which is very dangerous for the heart and liver due to the active polyphenol structure in curcumin, which exhibits iron chelating properties (Lee et al., 2016; Saeidnia et al., 2022).

This double-blind, randomized, controlled clinical trial with a reasonable sample size and longer intervention period was conducted to investigate how oral curcumin affected cardiac and hepatic T2*MRI and liver enzymes in patients with β‐thalassemia major.

## 2 Materials and methods

### 2.1 Study subjects

This double-blind, randomized was conducted from September 2021 to March 2022. A total of 171 patients over 5 years old with β‐thalassemia major were randomized in this study at Ali-asgar Hospital, Tehran, Iran, considering 20% dropouts in each group. All the patients were receiving deferasirox before initiating the study. At the beginning of the study, a checklist was provided in which information on demographic and basic clinical data of all patients, such as the age, alanine aminotransferase (ALT), aspartate transferase (AST), alkaline phosphatase (ALP), direct and total bilirubin, ferritin, and T2*MRI levels before and after treatment, were extracted.

The measurement of hemoglobin (Hb) was conducted with the utilization of an automated blood cell counter (Coulter Electronics, Ltd., United Kingdom). The concentration of magnesium was determined through the utilization of specialized kits designed for the assessment of each individual trace element (Biomed) by an automatic analyzer (Hitachi, Japan). The serum ferritin level was assessed by an enzyme‐linked immunosorbent assay method (Dade Behring, Inc., Newark, DE, United States). Serum ALP, ALT and AST levels were determined using a spectrophotometric method employing the Pars Azmun kit (Karaj, Iran). Serum total and direct bilirubin were measured photometrically based on diazo reaction and Pars Azmun kit (Karaj, Iran). The rate of hepatic hemosiderosis in patients was extracted according to the routine T2*MRI method (Siemens Healthineers, Germany).

The determination of the sample size was based on the primary outcome of a change in ferritin, according to previous studies (Moayedi et al., 2013). With the objective of achieving a confidence level of 95% and a power of 80%, the Pocock’s formula was employed to ascertain that a minimum of 60 patients per group would be necessary to attain an appropriate sample size. To account for a potential dropout rate of 30%, the sample size was subsequently increased to 80 in each group.

**FIGURE 1:**
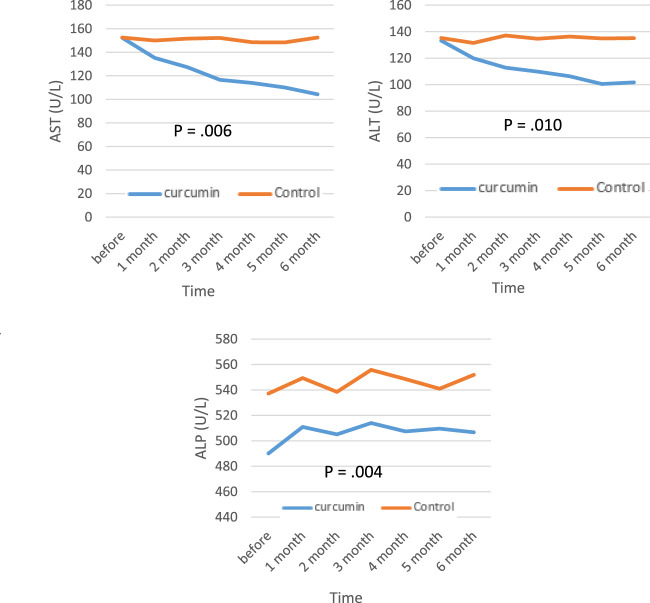
Flow chart of study procedure. ITT; intent-to-treat population.

Randomization (allocation ratio 1:1) was done by a biostatistician based on a simple randomization method using a computerized random number table inside the clinic. In this way, the patients were randomly divided into a curcumin-treatment group and a placebo group. The flowchart of the study is shown in Figure 1.

### 2.2 Inclusion and exclusion criteria

Inclusion criteria included the following: patients over 5 years of age with β‐thalassemia major who had liver enzymes higher than three times normal; transfusions beginning after 2 years of age; and starting iron chelator treatment before 5 years of age. Study exclusion criteria included patients with active infection, renal disease, uncontrolled diabetes, liver disease, inflammatory diseases, hepatitis B and C, human immunodeficiency virus, oral intolerance to the drug, cholecystectomy, splenomegaly, other hemoglobinopathies, patients without curcumin ingredient sensitivity, unwillingness to continue to participate in the study, and patients who refused to continue the study to the end.

### 2.3 Study intervention

In the curcumin-treatment group, patients received 500 mg oral curcumin capsules twice a day (1,000 mg/day) for 6 months (prepared by Aburaihan Pharmaceutical Company, Tehran, Iran). In the placebo group, patients received placebo capsules (starch) twice a day for 6 months (prepared by Aburaihan Pharmaceutical Company, Tehran, Iran). Curcumin and placebo were labeled by nurses with black and white labels, respectively, so researchers and study patients were blinded to treatment allocation during treatment and until the end of the study. Then, based on the randomization program, the nurse distributed the labeled capsules among the patients by assigning an identification code to each patient. Finally, based on the given identification codes, the results were collected and analyzed. The parents were requested to be careful about the use of medicines and not to stop taking them for any reason without the coordination of the doctor. Parents were also asked to record the number of supplements taken in order to determine the level of adherence to treatment. Patients were examined once a month for 6 months to receive capsules and measure the levels of ALT, AST, ALP, direct and total bilirubin, ferritin and T2*MRI.

### 2.4 Statistical analysis

Statistical analyses were performed using SPSS 25.0 software (Inc., Chicago, IL, United States). The normality of the variable distribution was determined by the Kolmogorov-Smirnov test. The results were presented as the mean ± standard deviation (SD) for numerical variables. Mean values of ALT, AST, ALP, bilirubin (direct and total), serum ferritin levels measured, and cardiac and hepatic T2*MRI before and after intervention were compared within groups using the paired *t*-test. An analysis of covariance was used to recognize any differences in cardiac and hepatic T2*MRI between the two groups after intervention, adjusting for baseline values and covariates. Statistical significance was defined as a *p*-value less than 0.05.

## 3 Results

A total of 158 patients with β-thalassemia major were analyzed in this study. Eighty-nine patients (56.3%) were male and 69 patients (43.7%) were female who had a packed red blood cell transfusion every 2–4 weeks. The mean ± SD age of the patients was 14.1 ± 11.8 years. None of the patients reported any specific side effects due to curcumin consumption, and all patients tolerated the capsules well in general. Mild gastrointestinal symptoms were reported in a few patients, which resolved spontaneously after a few days. From counting the number of capsules used, it was found that more than 92% of the capsules were used by the patients, which shows that the level of adherence to the treatment was acceptable.

There was no statistically significant difference between the study groups in the mean age, height, weight, body mass index, age of starting the transfusion, age at deferasirox onset, or gender (Table 1). The mean ± SD of the deferasirox receiving period was 11.1 ± 3.6 years. The mean serum ferritin levels was 1,418.2 ng/mL with a range from 351 to 6,236 ng/mL. The mean ± SD of Hb concentration was 7.9 ± 4.7 g/dL on average during the last 5 years. Based on the Hb concentration, all patients were transfused with 10–15 mL of packed red blood cells per kg of body weight.

**TABLE 1:** Demographic characteristics.

Characteristics	Curcumin group (N = 80)	Placebo group (N = 78)	*p-*_value_
Mean age ±SD, yrs	14.2 ± 10.0	13.9 ± 13.8	0.932^a^
Min-Max	8–21	7–23	
Gender, n (%)			0.525^b^
Male	43 (53.7)	46 (58.9)	
Female	37 (46.3)	32 (41.1)	
Mean weight ±SD, Kg	38.4 ± 20.5	32.2 ± 12.3	0.165^a^
Mean height ±SD, Cm	140.1 ± 18.7	135.8 ± 16.3	0.357^a^
Mean body mass index ±SD, Kg/m2	14.2 ± 10.0	13.9 ± 13.8	0.321^a^
Mean age of starting the transfusion, yrs	2.2 ± 3.1	1.9 ± 3.3	0.698^a^
Mean age at deferasirox onset, yrs	3.4 ± 3.7	3.8 ± 3.5	0.999^a^

SD; standard of deviation, n; number.

^b^ Pearsonʼs χ2 test was used.

^a^ Student t-test was used.

The mean values of serum magnesium and T2*MRI of patients with β‐ thalassemia major at baseline and after 6 months of intervention are presented in Table 2. No significant differences were observed between the curcumin and placebo groups at baseline values or at the end of the study of cardiac and hepatic T2*MRI or serum magnesium.

**TABLE 2:** T2*MRI of patients with β‐thalassemia major at baseline and after 6‐month intervention.

		Curcumin group (N = 80)		Placebo group (N = 78)		*p-*_value_ ^a^
		Before	After	Before	After	>0.05
Cardiac T2*MRI (ms) Hemosiderosis (n, %)	Mean ± SD	24.2 ± 9.3	22.1 ± 8.6	23.3 ± 10.2	22.7 ± 9.8	
	Normal (20≤)	48 (60)	50 (62.5)	42 (53.8)	43 (55.1)	
	Abnormal	32 (40)	30 (37.5)	36 (46.1)	35 (44.9)	
	Severe (9.99≥)	2 (2.5)	2 (2.5)	4 (5.1)	4 (5.1)	
	Moderate (10–13.99)	16 (20.0)	14 (17.5)	17 (21.8)	16 (20.5)	
	Mild (14–19.99)	14 (17.5)	14 (17.5)	15 (19.2)	15 (19.2)	
	Min	8.3	7.4	9.9	9.3	
	Max	39.8	37.7	40.7	39.5	
	*p-*_value_ ^b^	>0.05		>0.05		
Hepatic T2*MRI (ms) Hemosiderosis (n, %)	Mean ± SD	7.8 ± 6.2	6.9 ± 5.3	8.3 ± 5.5	8.1 ± 4.4	>0.05
	Normal (6.3≤)	14 (17.5)	18 (22.5)	19 (24.4)	21 (26.9)	
	Abnormal	66 (82.5)	62 (77.5)	59 (75.6)	57 (73.1)	
	Severe (1.39≥)	6 (7.5)	5 (6.2)	8 (10.2)	8 (10.2)	
	Moderate (1.4–2.79)	26 (32.5)	22 (27.5)	20 (25.6)	18 (23.1)	
	Mild (2.8–6.29)	34 (42.5)	35 (43.7)	31 (39.7)	31 (39.7)	
	Min	1.5	1.2	1.9	1.6	
	Max	32.3	30.4	35.4	34.1	
	*p-*_value_ ^b^	>0.05		>0.05		
**Serum magnesium (mg/dL)**	Mean ± SD	1.7 ± 0.3	1.3 ± 0.5	1.9 ± 0.8	1.6 ± 0.4	>0.05
	Min	1.5	1.1	1.8	1.6	
	Max	2.4	2.0	2.9	2.7	
	*p-*_value_ ^b^	>0.05		>0.05		

MRI: magnetic resonance imaging; ms: milliseconds; SD: standard of deviation; SD; n; number.

^b^ *p* value is reported based on the analysis of paired sample *t*-test.

^a^ *p* value is reported based on the analysis of covariance.

**FIGURE 2:**
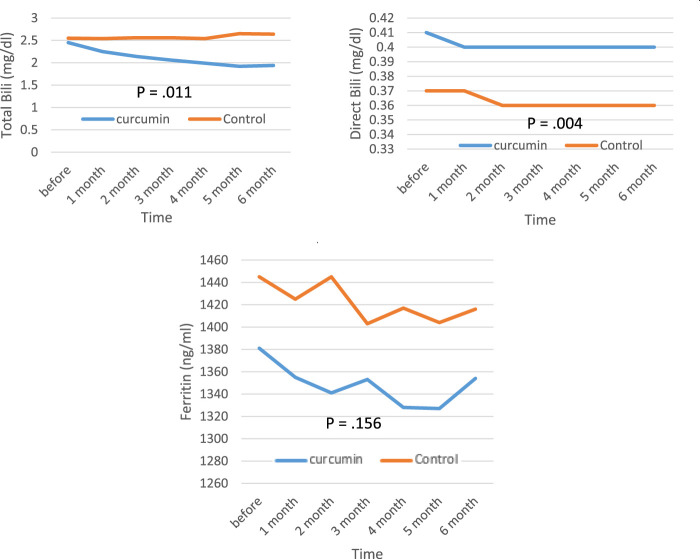
Mean values of ALT, AST, ALP, bilirubin (direct and total), and serum ferritin levels before and after intervention in the studied groups. ALT; alanine aminotransferase, AST; aspartate transferase, ALP; alkaline phosphatase.

The biochemical parameters of patients with β‐thalassemia major are presented in Figure 2. From the results of the analysis of covariance, there was a significant decrease in levels of AST, ALT, ALP, and bilirubin (direct and total) in the curcumin group compared with the placebo group by the end of the study (*p* < 0.05). The levels of serum ferritin remained unchanged in both groups at the end of the follow‐up period (*p* > 0.05).

The statistical analysis of the data revealed that the mean of ALT and AST before the intervention was the same in the two groups and also, in the assessment of the first month after the intervention, there was not much difference between the two groups, but in the second, third, fourth, fifth, and sixth months after the intervention, the curcumin group was much lower than the control group, and this difference increased every month. The mean of ALP was higher in the control group before the intervention, but after the intervention, it began to decrease in the curcumin group, and finally, 3 months after the intervention, it was lower in the curcumin group than in the control group. The mean of total bilirubin was the same in both groups before the intervention, but it started to decrease in the second month, and this trend continued until the end of the sixth month. The mean of direct bilirubin, ferritin level and the mean of T2*MRI (the amount of iron overload) did not find any significant difference in the two groups until the end of the study.

## 4 Discussion

The clinical evidence has clearly indicated that curcumin possesses a robust capacity to provide protection against conditions such as cancer, inflammation, as well as neurological disorders (Al- et al., 2016; Nasseri et al., 2018). In the present study, it was observed that curcumin supplementation significantly reduced ALT, AST, ALP, and bilirubin (direct and total) levels in these patients.

There exists a paucity of evidence regarding the impacts of curcumin on the surplus of iron in individuals suffering from β‐thalassemia. In the only available report, a proteomics technique was employed to ascertain the protein arrangement and oxidative impairment within the plasma of ten β‐thalassemia patients, both prior to and after the administration of curcumin. Daily administration of curcumin at a dosage of 500 mg over a period of 12 months in the aforementioned research yielded a noteworthy decrease in non-transferrin-bound iron, an index of free iron (Weeraphan et al., 2013).

Alizadeh et al. conducted an animal study and divided 50 Wistar rats into three groups. Similar to the findings of our study, they found that curcumin significantly reduced the levels of ALT, AST, and antioxidants in the blood (Özbolat and Yegani, 2021). Curcumin also caused a significant decline in liver enzymes and cholesterol levels (Panahi et al., 2016; Rahmani et al., 2016). Contrary to the results of this study, Saadati and others demonstrated that curcumin at a 1,500 mg dosage had no significant impact on liver variables, including ALT and AST compared to placebo (Saadati et al., 2019). Similarly, a meta-analysis of four randomized clinical trials including 228 patients revealed a significant decrease in circulating ALT and AST, and bilirubin concentrations with a daily intake of 1,000 mg of curcumin in 8 weeks (Mansour-Ghanaei et al., 2019). In our investigation, after 6 months of assessment, neither the serum ferritin level nor the amount of iron overload determined by T2MRI significantly decreased. In contrast, after 3 months of consuming 1,500 mg of curcumin daily, serum iron, transferrin saturation, and ferritin levels decreased significantly compared to placebo in several investigations (Ghone et al., 2008; Weeraphan et al., 2013; Saeidnia et al., 2022). Additionally, a decline in free iron index and serum non-transferrin binding iron was observed in another study, while a decline in total iron binding capacity and ferritin levels was not (Mohammadi et al., 2018).

The discrepancy in the findings of the aforementioned studies is likely to have arisen from variations in the clinical attributes of the subjects, the dosage of curcumin administered, the duration of the intervention, and the methodology employed in the research. Furthermore, the outcomes of the study may have been influenced by the formulation of the supplement. It has been proposed that the bioavailability of native curcumin is diminished due to its hydrophobic nature, whereas certain researchers contend that the utilization of nanoparticle formulations enhances both the bioavailability and therapeutic efficacy of curcumin.

According to previous reports, curcumin reduces iron overload by directly interacting with iron via the beta diketone group and altering the expression of proteins involved in iron metabolism, such as hepcidin (Benassi et al., 2007; Jiao et al., 2009). Differences in study results may be due to the clinical characteristics of the participants, curcumin doses, or the duration of the intervention. Additionally, the formulation of curcumin utilized might have an impact on the study’s findings.

Curcumin has limited bioavailability due to its hydrophobic nature, so some studies have claimed that the formation of curcumin nanoparticles increases curcumin’s bioavailability and therapeutic efficacy (García-Niño and Pedraza-Chaverrí, 2014). Also, curcumin, like other low-molecular-weight flavonoids, may act synergistically with iron chelators to reduce iron overload, but its exact function is debatable (Fibach and Rachmilewitz, 2010). Curcumin exhibits a broad range of pharmacological activities, including anti-inflammatory, antioxidant, anticancer, antimicrobial, reduction of blood fat, inhibition of lipoxygenase, liver protection, inhibition of cyclooxygenase, removal of free radicals, inhibition of proteases, inhibition of fat oxidation, reduction of platelet aggregation, reduction of cholesterol, and reduction of proliferation (Ono et al., 2004; Maheshwari et al., 2006; Sahu et al., 2008; Modaresi et al., 2017). Typically, inflammation and oxidative stress play a significant role in the progression of liver cell damage. Because of the antioxidant and anti-inflammatory effects of curcumin, it prevents liver cell damage. Curcumin has also been identified as a potent anti-fibrosis drug by increasing the production of matrix metalloproteinases enzymes in the liver (Fu et al., 2008; Lee et al., 2016; Rukkumani et al., 2004).

The level of liver enzymes in this study decreased after 1 month of curcumin intake due to its anti-inflammatory, anti-fibrogenic, and antioxidant activities, and 3 months later, the level of humeral alkaline phosphatase showed a declining trend consistent with the most recent animal studies (Dalvand and Behpoor, 2019; Khezri Motlagh et al., 2021) and human studies (Maghsoumi and Arbabi Bidgoli, 2019; Pakzad et al., 2019; Nazarieh et al., 2020).

However, there were some limitations in the present research. Although the participants were instructed to maintain their usual dietary patterns throughout the duration of the research, there was a lack of precise data regarding the consumption of curcumin among the subjects. Furthermore, the Nutritionist 4 software did not provide a specific coding system for spices. Additionally, it would be beneficial to assess additional markers of iron status, such as ferroportin and erythropoietin, in order to enhance the interpretation of the findings.

## 5 Conclusion

Administration of curcumin has some beneficial effects on liver function by reducing liver enzymes in patients with beta-thalassemia major.

## Data Availability

The raw data supporting the conclusions of this article will be made available by the authors, without undue reservation.
